# Diabetes and its Potential Impact on Head and Neck Oncogenesis

**DOI:** 10.7150/jca.35607

**Published:** 2020-01-01

**Authors:** Xiaofeng Wang, Huiyu Wang, Tianfu Zhang, Lu Cai, Enyong Dai, Jinting He

**Affiliations:** 1 Department of Stomatology, China-Japan Union Hospital of Jilin University, Changchun 130033, Jilin Province, China; 2 Pediatric Research Institute, Department of Pediatrics, The University of Louisville School of Medicine, Louisville, KY 40292, USA; 3 Departments of Radiation Oncology, Pharmacology, and Toxicology, University of Louisville, Louisville, KY 40202, USA; 4 Department of Neurology, China-Japan Union Hospital of Jilin University, Changchun 130033, Jilin Province, China; 5 Department of Oncology and Hematology, China-Japan Union Hospital of Jilin University, Changchun 130033, Jilin Province, China

**Keywords:** hyperglycemia, insulin resistance, hyperinsulinemia, chronic inflammation, immune system dysfunction, metformin

## Abstract

In recent years, the incidence of diabetes mellitus and cancer has increased sharply; indeed, these have become the two most important diseases threatening health and survival. Head and neck (HN) tumors are the sixth most common malignancies in humans. Numerous studies have shown that there are many common risk factors for diabetes mellitus and HN squamous cell carcinoma, including advanced age, poor diet and lifestyle, and environmental factors. However, the mechanism linking the two diseases has not been identified. A number of studies have shown that diabetes affects the development, metastasis, and prognosis of HN cancer, potentially through the associated hyperglycemia, hyperinsulinemia and insulin resistance, or chronic inflammation. More recent studies show that metformin, the first-line drug for the treatment of type 2 diabetes, can significantly reduce the risk of HN tumor development and reduce mortality in diabetic patients. Here, we review recent progress in the study of the relationship between diabetes mellitus and HN carcinogenesis, and its potential mechanisms, in order to provide a scientific basis for the early diagnosis and effective treatment of these diseases.

## Introduction

“Head and neck” (HN) tumors include any tumor occurring in the oral cavity, oropharynx, hypopharynx, larynx, and esophagus. More than 90% of these are squamous cell carcinomas (SCCs) 1. HN neoplasia is a common disease that seriously threatens human health, with 550,000 new cases and 300,000 deaths being reported annually worldwide during the past 30 years 2. Furthermore, in 2019 it was estimated that there were 53,000 new cases of oral cavity or pharyngeal tumor and 10,860 deaths from these annually in the USA alone 3. Oral malignant tumor is the most common malignant tumor of the head and neck, and >90% of these are SCCs, such that its incidence is ranked sixth among all the malignant tumors 4.

Diabetes mellitus (DM) is a group of metabolic diseases characterized by hyperglycemia and caused by insufficient insulin secretion and/or defective insulin action 5. In developed countries, the prevalence of diabetes has reached 3-7%, and it has become the fifth leading cause of death worldwide, next only to cancer, acquired immune deficiency syndrome, and cardiovascular disease 6-10. A previous report shows that the number of diabetic patients worldwide reached 382 million in 2014; this number is expected to reach 592 million by 2035 11.

Type 1 diabetes was previously termed “insulin- dependent DM” or “juvenile DM”. Type 2 diabetes mellitus (T2DM), previously termed “non-insulin- dependent DM”, is characterized by insulin resistance, which implies that target cells are unable to respond appropriately to insulin, and this form of the disease accounts for more than 90% of DM cases 12. A substantial amount of epidemiological evidence indicates that diabetes not only increases the risk of many types of cancers, but also affects the long-term efficacy of cancer treatment. As early as 1932, Wilson 13 noticed that patients with T2DM may be at greater risk of tumor development. In recent years, a large number of studies have shown that diabetes significantly increases the incidence of malignant tumors and affects therapeutic efficacy 14. It has been reported that diabetes is more common in patients with breast cancer, colorectal cancer, uterine cancer, liver cancer, and pancreatic cancer 15-24. However, there is little or no information about how diabetes impacts HN and particular oral cancers. Here, we review available related studies and identify certain links between diabetes and HN cancers. We conclude that when diagnosing diabetes, additional attention should be paid to the possibility of co-existing malignant tumors. Emerging evidence indicates that metformin prevents not only diabetic cardiovascular complications, but also reduces tumor incidence and progression.

## DM and HN tumors

Maynard and Pearson first identified a link between DM and tumorigenesis in 1909 25. Epidemiologic studies have since shown that diabetic patients are at greater risk of colon cancer, endometrial cancer, breast cancer, and others 26-28. This phenomenon is particularly evident with respect to colon cancer, with clinical studies having shown that the risk of colon cancer in diabetic patients is 1.3-3.0 times higher 28-30. As a consequence, the American Oncology Association and the American Diabetes Association issued a statement regarding the possible links between cancer and diabetes (principally T2DM), which was divided these into three categories: (a) unmodifiable risk factors (age, sex, and ethnicity), (b) modifiable risk factors (including obesity, physical activity, smoking, and alcohol consumption), and (c) biological links between diabetes and cancer (for example, hyperinsulinemia, hyperglycemia, insulin resistance, and chronic inflammation) 31.

There are many common risk factors for DM and HNSCC, including advanced age, poor diet and lifestyle, and environmental factors 32. In Asia, Tseng found a higher risk of HN cancer in individuals with DM than in those without 33, and a retrospective study of patients with oral SCC (n=600) and controls (n=574) found that there was a higher prevalence of DM in the former (24.3% *versus* 11.1%) 34. Recently, it has been shown that the overall survival, relapse-free survival, and tumor-specific survival of patients with oral SCC and DM are lower than in patients without DM, and the general prognosis is also poorer 35. An estimated 30.3 million people of all ages (or 9.4% of the U.S. population) had diabetes in 2015^36^. Furthermore, Bányai *et al*. demonstrated a correlation between oral cancer and metabolic disorders (DM and fasting hyperglycemia) in Hungary, with the number of people having these defects being much larger among patients with oral SCC than among controls 37. Bao *et al*. examined data from 25,154 twins born until 1958 in the Swedish Twin Registry to determine whether diabetes in middle age affects the risk of cancer in later life, and whether genetic and early-life environmental factors might play a role in any association, and found that DM does indeed increase the risk of pharyngeal cancer in later life 38. Another study suggested that patients with DM are at significantly greater risk of thyroid cancer than non-diabetic individuals, although, whereas there was a strong positive association in women, it was not significant in men 39. Liu *et al*. found that long-term exposure to high glucose concentrations predisposes to the development of malignant tumors *in vitro* and *in vivo*. Compared with non-diabetic patients with HN tumors, those with DM had a lower survival rate, suggesting that diabetic pathophysiology may have a significant influence on HN SCC metastasis. 40. In addition, a number of epidemiologic studies have shown that DM increases the risk of oral tumors and precancerous lesions, such as leukoplakia, erythema, and lichen planus 34,41-43. By contrast, Stott-Miller *et al*. found a weak negative correlation between DM and HNSCC (odds ratio, 0.92; 95% confidence interval, 0.88-0.96), although this is not consistent with the majority of research findings 44. Hence, we first summarize the link between DM and HN tumorigenesis (a brief overview is presented in Table 1).

Western and Asian diets contain different nutrients, which may have different effects on development of diabetes. In the U.S., Ankola *et al* 45 confirmed that from 2002 to 2011, HNSCC patients at Montefiore Medical Center in the Bronx (NY) comprised those with primary oropharyngeal (36%), laryngeal (33%), and oral cavity cancers (31%). In Japan, Nishimura *et al* 46 examined 107 HN cancer patients from January 2004 to August 2005 and found that the incidence of end-stage renal disease is 1.3 times higher in the United States than in Japan. In Taiwan, Tseng found that the incidence of HNC in patients newly diagnosed with DM was 1.47 times higher than that in a control group 33.

Although many studies have demonstrated a relationship between DM and HN tumors, the mechanisms responsible for this link are not yet understood. DM may affect the development, metastasis, and/or prognosis of HN cancer through the effects of hyperglycemia, hyperinsulinemia, insulin resistance, chronic inflammation, and/or microvascular disease, which may be negatively correlated with the prognosis of the tumor 34,35,47. Furthermore, anti-hyperglycemic drugs with differing mechanisms of action may have differential effects on oral SCC. Therefore, we next review the effects of DM and anti-hyperglycemic drugs on oral SCC and the potential mechanisms involved.

## Potential mechanisms whereby DM influences HN tumors

Tumor formation is a complex process, but it can be divided into initiation, promotion, and progression phases, and various factors can influence each^48^. When the complexity of the pathogenesis of DM is added, it becomes clearer why the relationship between DM and cancer has yet to be fully explained 49. However, DM may affect the development of oral tumors through the influence of pathologic features, including hyperglycemia, hyperinsulinemia, insulin resistance, chronic inflammation, and immune system dysfunction.

### Hyperglycemia

It is well known that cancer cells show more rapid glucose uptake and metabolism, i.e. increased expression of glucose transporter 1 (GLUT-1) and HK-II, than normal cells; these characteristics enable rapid growth and division^50^. Hyperglycemia and hyperlipidemia are the main pathologic features of the metabolic disorders that comprise DM, and hyperglycemia implies the availability of an of the most important metabolic substrate for tumor cells 51. Hyperglycemia promotes cellular proliferation by increasing expression of GLUT-1, GLUT-3, protein kinase C, peroxisome proliferator-activated receptor alpha/gamma and epithelial growth factor 52. *In vitro* experiments have shown that high glucose concentrations damage non-tumor cells and lead to many diabetic complications 53. By contrast, most cancer cells are highly dependent on glucose and persistently high glucose concentrations can promote the growth of cancer cells 54. Furthermore, a previous study found that hyperglycemia is a risk factor for gingival cancer, which may be due to a reduction in the bactericidal activity of immune cells in a high glucose environment, increasing susceptibility to bacterial infection 55. IGF-1 is expressed by oral SCCs 56; therefore, there may be a risk that hyperglycemia increases proliferation of oral SCC cells.

However, hyperglycemia is not only able to stimulate proliferation of cancer cells; it can also induce tumor resistance to chemotherapy. For instance, persistent high glucose concentrations protect HN SCCs by reducing the cytotoxic effect of cisplatin, which may be the result of higher expression of the *Abcg2* gene, a mediator of drug resistance 57.

### Hyperglycemia and obesity

With the improvement in living standards, the associated consumption of high-energy diets has increased the prevalence of obesity. Obesity is associated with a greater risk of cardiovascular and cerebrovascular disease, and there is evidence that obesity is also a risk factor for malignancy. Previous studies suggest that the high incidence of malignant tumors in obese patients is related to the associated reduction in adiponectin secretion, the increase in leptin secretion, and the higher insulin-like growth factor (IGF) expression. Recent studies have elucidated the relationship between obesity and malignancy from a number of perspectives. Obesity results in tissue hypoxia, expression of tumor-susceptibility genes, and a higher rate of differentiation of adipose stromal cells, which promote the transformation of normal cells into malignant tumors 58. Hyperlipidemia is a risk factor for oral cancer 33. In addition, obese and diabetic Tsumura Suzuki Obese Diabetic mice are susceptible to 4-nitroquinoline 1-oxide-induced esophageal carcinogenesis, suggesting that obesity and DM are risk factors for esophageal SCC 32. Dysregulation of 72 lipid metabolites has been demonstrated in oral SCC, and a combination of TGFB1 (transforming growth factor-β1), SPP1 (secreted phosphoprotein-1), and SERPINNE1 (Serine protease-1) is useful for predicting oral SCC prognosis 59.

### Insulin resistance, hyperinsulinemia, and IGFs

Hyperinsulinemia, which develops secondary to insulin resistance, plays an important role in the development of malignant tumors. When target cells fail to respond effectively to normal insulin concentrations, pancreatic insulin secretion increases in compensation, leading to hyperinsulinemia, which can affect cell proliferation and metabolism, and promote tumor formation. Indeed, it has been reported that insulin resistance and hyperinsulinemia directly promote carcinogenesis in DM patients 60,61. A previous meta-analysis shows that hyperinsulinemia is a risk factor for multiple malignant tumors 62.

Furthermore, not only insulin itself, but also the insulin receptor, IGF receptor, and IGF, play roles in tumorigenesis. Oxidative stress is a key mechanism of insulin resistance, with large quantities of superoxide anions being produced in mitochondria, which inactivate insulin receptors and are carcinogenic 63. Insulin can increase the concentration of free, biologically active IGF-1 in the circulation by inhibiting the synthesis of IGF-binding protein in the liver 64. Human cancer cells generally express both the insulin receptor and the IGF-1 receptor 65. Activation of insulin receptors can induce mitosis 66, so it may be that insulin can promote the development and progression of HN tumors, but insulin may also have an indirect tumorigenic effect through IGF-1 67. Indeed, IGF-1 has been shown to be a more potent promoter of cell mitosis and inhibitor of apoptosis than insulin 68. Consequently, a high concentration of IGF-1 suppresses apoptosis and induces cell cycle progression, angiogenesis, and metastatic activity in various cancers 69. Meanwhile, studies have shown that insulin receptors and IGF receptors are highly expressed in many tumors 49,70,71. IGF-1, acting through its receptors, also promotes neovascularization by inducing vascular endothelial growth factor gene transcription, which promotes tumor development 72. Binding of insulin or IGF-1 to their receptors activates many signaling pathways, which can promote the proliferation, invasion, and metastasis of cancer cells, inhibit apoptosis, and lead to the development and progression of many types of cancer cell 73. In particular, binding of either ligand to a receptor activates the phosphoinositol 3-kinase/Akt/ mechanistic target of rapamycin (mTOR) pathway, leading to abnormal cell proliferation, inhibition of apoptosis, and carcinogenesis 74. Importantly, IGF-1 is expressed in oral SCCs 56.

### Chronic inflammation

Pro-inflammatory cytokines, such as interleukin (IL)-1, IL-6, and tumor necrosis factor alpha (TNF-alpha), can cause abnormal serine phosphorylation of insulin receptor substrates through multiple pathways, which inhibits normal tyrosine phosphorylation, thereby interfering with insulin signal transmission and inducing insulin resistance 75. Fewer tumor suppressor and larger numbers of pro-inflammatory cells are found in oral and esophageal tumors 76-78. Furthermore, the infiltration of inflammatory cells can increase the risk of cancer by secreting bioactive molecules, such as cytokines and chemokines 79. In addition, the production of anti-inflammatory cytokines is impaired, complement-mediated phagocytosis is inhibited, and chemotactic phagocytosis by monocytes/macrophages is impaired in DM patients 80, leading to a higher probability of infection. Consistent with this, epidemiologic studies have shown that cancer is often associated with chronic inflammatory disease and infection. Inflammation can play a role in various stages of cancer development through a number of mechanisms 81. Hyperglycemia-induced chronic inflammation is very common in the tissues of diabetic patients, and in particular, T2DM is associated with a variety of oral inflammatory diseases 82,83. Furthermore, there is evidence 84-87 that persistent inflammation increases genetic instability and cancer risk. Chronic inflammation is associated with a high concentration of TNF-alpha, which activates the mitogen-activated protein kinase (MAPK) and nuclear factor-kappa B (NF-κB) signaling pathways. NF-κB not only promotes the proliferation of malignant cells and inhibits apoptosis, thereby improving their survival rate, but also promotes angiogenesis and metastasis. In these ways, it can mediate the responses of cells to hormones and chemotherapeutic drugs 84,88. Various chronic inflammatory diseases have been shown to accelerate carcinogenesis. Atrophic stomatitis and the metabolic and immunologic changes in DM lead to cellular transformation and promote the development of precancerous lesions, such as leukoplakia. *Candida* infection is highly prevalent in DM patients, and can induce squamous epithelial metaplasia and epithelial cell proliferation 89. Finally, a recent study that investigated the effects of DM on the activation of signal transduction pathways in oral cancer found that DM accelerates cellular proliferation by activating the Ras/Raf/ MAPK signal transduction pathway 90.

Diabetic patients are susceptible to infection because high blood sugar levels promote survival of bacteria. An etiological study of HNSCC found that human papillomavirus (HPV) infection was significantly associated with occurrence and development of HN tumors 91. More and more studies show that high-risk HPV 16/18 subtype infection is another key independent pathogenic factor for HNSCC (after smoking and drinking), especially in those with oropharyngeal cancer 92. In HNSCC, HPV mainly infects basal epithelial cells and mucosal cells. HPV integrates ribonucleic acid into host cells and induces deletion of E2 gene fragment. The E2 gene negatively regulates HPV-E6/HPV-E7 oncogenes 93. In cases where E2 deletion leads to increased expression of HPV-E6/HPV-E7 genes, the expressed proteins can bind and degrade P53 and pRb, respectively, meaning that infection is not detrimental to the host. Dysregulation of the cell cycle leads to infinite proliferation and malignant transformation of host cells 94. Surviladze et al. 95 showed that HPV 16 virus inhibits autophagy in host cells via the P13K/mTOR signaling pathway, thereby affecting the cell cycle.

### Immunologic system

Cellular immunity, humoral immunity, and cytokines all play a role in anti-tumor immunity, but cellular immunity is the most significant. Their poor immune function and hyperglycemia make diabetic patients more susceptible to infection and make such infections more difficult to control. Cellular immune dysregulation reduces anti-tumor immunity 96-98. For example, hyperglycemia affects the function of B lymphocytes, which produce antibodies in response to antigen stimulation, and low levels of immunoglobulin (Ig)M, IgA, and IgG indicate humoral immune deficiency 99. T lymphocytes are classified into cluster of differentiation (CD)8+ and CD4+ subgroups, but CD3+ cells are mature T lymphocytes, meaning that a reduction in peripheral CD3+ cell numbers is consistent with a higher degree of T lymphocyte immaturity 100,101. Such immune deficiency increases susceptibility to infection 102,103. However, the immune surveillance systems of the body cannot detect transformed tumor cells, permitting the development of malignant tumors.

Oral lichen planus (OLP) is a precancerous lesion, and the relationship between DM and OLP has been the subject of significant research. It has been suggested that the endocrine dysfunction in DM and the related immunodeficiency may contribute to the development of OLP 43. Epidemiologic studies have supported links between DM and oral precancerous lesions, such as mucosal leukoplakia 41, erythema 42, and lichen planus 43. These may be mediated by immune deficiency, secondary to endocrine dysfunction, which accelerates the development of such lesions.

## Effect of metformin and other diabetic drugs on HN tumors

Metformin is a biguanide drug that inhibits hepatic gluconeogenesis and increases tissue insulin sensitivity, thereby having an anti-hyperglycemic effect 104. It is well tolerated, and its pharmacokinetics are well characterized 104,105. It was originally extracted from *Syringa franca* and is now used as the first-line drug for the treatment of T2DM, especially in the presence of obesity 106. Interestingly, there is growing evidence that metformin also has antineoplastic effects. Its use is associated with a significant reduction in the risk of cancer and improvements in the survival of diabetic patients with medullary thyroid carcinoma 107, esophageal cancer 108, lung cancer 109, liver cancer 110, gastric cancer 111,112, pancreatic cancer 113,114, colorectal cancer 115, prostate cancer 116,117, ovarian cancer 118, endometrial cancer 119, and breast cancer 120.

There has also been significant research into the potential of metformin for the prevention and treatment of HN cancer, which is summarized in Table 2. Rêgo *et al*. systematically reviewed the effects of metformin in patients with HN SCC before 2014, finding that metformin reduces the local recurrence and metastasis of such tumors, and improves overall survival and disease-free survival 121. Skinner *et al*. found that patients treated with metformin had a significantly lower incidence of local recurrence after radiotherapy for HN SCC: their overall 5 year survival was 2.1 times that of patients not treated with metformin. However, the report did not indicate whether these patients had DM 122. Yen *et al*. studied the risk of HN SCC in 66,600 diabetic patients in Taiwan, and found that the incidence of HN SCC was 0.34 times lower when metformin was being administered than when it was not, although the benefit was clearest for patients over 40 years of age^123^. In 2014, Becker *et al*. found no significant difference in the risk of HN SCC between patients taking metformin and those who were not, but also found that long-term use of metformin reduced the risk of laryngeal cancer^124^. Kwon *et al*. found that diabetic patients with HN SCC who were not using metformin demonstrated a higher incidence of recurrence than non-diabetic patients^125^. And in 2016, Figueiredo *et al*. found that diabetic patients had a lower risk of HN SCC than non-diabetic patients. Therefore, it was suggested that the reason why the presence of DM is associated with a lower risk of HN SCC might be due to the use of metformin^126^.

To investigate this possibility, Vitale *et al*. used the carcinogen 4-nitroquinoline 1-oxide to induce tongue cancer in mice, and found that metformin prevented development of this form of HNSCC^127^. Patel *et al*. found that OCT-3 (octamer-binding transcription factor 3) is not expressed in normal oral cells, but is expressed in HN SCCs. Metformin inhibited high expression of OCT-3 in HN SCCs, but had little effect on low levels of expression. When expression or activity of OCT-3 was blocked, metformin could not induce AMP-activated protein kinase (AMPK) activation and inhibit mTORC1^128^. Madera *et al*. reached similar conclusions^129^. A retrospective study published by Stokes *et al*. 130 in 2018 found that, HN cancers patients with DM improved greatly after taking metformin. These results suggest that metformin can improve the cancer-specific survival rate in patients with both HN cancer and DM. In addition, another recent study demonstrated that metformin suppresses proliferation of human SCC FaDu cells by reducing SNHG7( small nucleolar RNA host gene 7) expression *via* SAHH-mediated DNA methylation 131. Kuo *et al*. suggested that metformin inhibits proliferation of non-stem HN SCC cells, but that it also causes a dose-dependent induction of the stem cell genes CD44(cluster of differentiation 44), BMI-1( B-lymphoma Moloney murine leukemia virus insertion region-1), and OCT-4(octamer-binding transcription factor 4) to protect SCCs against the effect of cisplatin *in vitro* 132.

Some epidemiological studies show that sulfonylureas increase the risk of cancer in those with T2DM. One possible mechanism is based on the fact that individuals with T2DM are insulin-resistant; sulfonylureas promote insulin secretion, thereby increase circulating insulin levels even further 133. A Meta-analysis by Thakar *et al.* 134 on the relationship between use of metformin and sulfonylureas and the risk of tumorigenesis in T2DM patients showed that metformin reduced the risk of tumorigenesis whereas sulfonylureas may increase the risk of tumorigenesis. In addition, based on adverse events reported by FDA (Food and Drug Administration of the United States) analysis data, use of the GLP-1 receptor agonist exenatide and the DPP-4 inhibitor statin has been questioned 135; these two drugs may increase the risk of malignant tumors, especially thyroid cancer and pancreatic cancer.

Although numerous studies of the anti-tumor effects of metformin and other diabetic drugs have been conducted, the mechanism involved remains unclear. Insulin resistance and hyperinsulinemia can directly promote carcinogenesis in diabetic patients, and metformin reduces cancer risk by ameliorating insulin resistance, hyperglycemia, and hyperinsulinemia 60, 61. However, other anti-diabetic drugs, such as pioglitazone, also ameliorate insulin resistance but do not have significant effects on cancer 47. Therefore, metformin may have a specific additional anti-cancer property 47. The mechanisms involved in the anti-tumor effect of metformin may include activation of the AMPK pathway 136-139, the induction of cell cycle arrest 140,141, the promotion of cell apoptosis 136,142, the inhibition of the invasion and metastasis of cancer cells 143,144, and the killing of cancer stem cells 143.

## Conclusions and perspectives

Both DM and malignant tumors are highly prevalent, and their incidences are increasing year by year 145. They are therefore both major diseases threatening human health, but there are numerous and varied uncontrollable risk factors for each, and differences in the research populations, sample sizes, and statistical methods used in different studies complicate their identification. Thus, whether DM is a risk factor for HN tumors or affects their prognosis remains uncertain. Many studies have shown that there is a relationship between the two diseases, but the mechanism involved is clearly complex and requires further elucidation. Recent studies have shown that DM may be associated with higher malignancy of HN tumors, through hyperglycemia, insulin resistance, chronic inflammation, and/or immune deficiency, which can seriously affect the quality of life of patients with HN tumors. Furthermore, although DM is easily diagnosed, the diagnosis of malignant tumors often requires the use of invasive diagnostic procedures. Nevertheless, it seems clear that diabetic patients have a higher risk of malignant tumors than the general population, implying that, when diagnosing diabetes, attention should be paid to the possibility of malignant tumors also being present. Furthermore, the use of metformin as the first-line medication for T2DM is also associated with an anti-tumor effect. We believe that further research may support the production of a new generation of anti-cancer drugs based upon metformin.

## Figures and Tables

**Table 1:** Link between diabetes mellitus and head and neck tumorigenesis

Cancer type	Findings	Reference
Head and neck squamous cell carcinoma (HNSCC)	Incidence of oral cavity cancer, oropharyngeal cancer, and nasopharyngeal carcinoma were significantly higher in patients with DM aged 40 to 65 years.	32
Head and neck squamous cell carcinoma (HNSCC)	Long-term exposure to high glucose concentrations predisposes to the development of malignant tumors *in vitro* and *in vivo*.	40
Pharyngeal cancer	DM does indeed increase the risk of pharyngeal cancer in later life	38
Thyroid cancer	Women patients with DM are at significantly greater risk of thyroid cancer than non-diabetic individuals.	39
Oral tumors and precancerous lesions	DM increases the risk of oral tumors and precancerous lesions, such as leukoplakia, erythema, and lichen planus.	33,41-43
Oral squamous cell carcinoma (OSCC)	OSCC Patients with DM had a significant increase in mortality and recurrence compared with those without DM.	34

**Table 2:** Published studies regarding the effects of metformin on head and neck tumors

Year	Author (Ref.)	Principal Findings
2012	Skinner *et al*. (122)	Metformin reduces the incidence of local recurrence after radiotherapy.
2012	Vitale *et al*. (127)	Metformin prevents development of tongue tumors induced by 4-nitroquinoline 1-oxide.
2013	Patel *et al*. (128)	Metformin inhibits the activity of OCT-3-expressing head and neck squamous cell carcinomas.
2014	Yen *et al*. (123)	The incidence of head and neck squamous cell carcinoma was lower when metformin was used in patients over 40 years old than when metformin was not used.
2014	Becker *et al*. (124)	Long-term use of metformin reduces the risk of laryngeal cancer.
2015	Rêgo *et al*.(121)	Metformin reduces the local recurrence and metastasis of head and neck squamous cell carcinoma.
2015	Kwon *et al*. (125)	Diabetic patients with head and neck squamous cell carcinoma who did not use metformin had a higher incidence of recurrence than non-diabetic patients.
2015	Madera *et al*. (129)	Metformin prevents tumor growth in HNSCC cells oncogenes expressing OCT-3 driven by mutant PIK3CA and HPV inhibiting mTOR signal.
2016	Figueiredo *et al*. (126)	People with diabetes have a lower risk of HNC than those without diabetes; metformin use may explain this inverse association.
2018	Stokes *et al*. (130)	Metformin improves the cancer-specific survival rate in patients with head and neck cancer and diabetes mellitus.
2019	Ping Wu *et al*. (131)	Metformin suppresses FaDu cell proliferation by reducing SNHG7 expression via SAHH-mediated DNA methylation.
2019	Kuo *et al*. (132)	Metformin inhibits the proliferation of non-stem HN SCC cells, but causes a dose-dependent induction of the stem cell genes CD44, BMI-1, and OCT-4 to protect SCCs against cisplatin *in vitro*.

OCT-3: octamer-binding transcription factor 3; OCT-4: octamer-binding transcription factor 4; PIK3CA: phosphatidylinositol-3-kinase catalytic subunit alpha; HPV: human papillomavirus; CD44: cluster of differentiation 44; BMI-1: B-lymphoma Moloney murine leukemia virus insertion region-1.
